# Hospital and Institutional News

**Published:** 1912-01-13

**Authors:** 


### HOSPITAL AND INSTITUTIONAL NEWS.

### THE NEW LUNACY COMMISSIONERS.

The Lord Chancellor has appointed Dr. Charles Hubert Bond, M.D., Medical Superintendent of the Longgrove County of London Mental Hospital, and Mr. B. T. Hodgson, barrister and secretary to the Commission, to be Commissioners in Lunacy. The new secretary in place of Mr. Hodgson is Mr. Oswald Eden Dickinson, barrister, who has acted as secretary to the Lord Chancellor's Visitors in Lunacy. It should be remembered that the Lunacy Act passed in the last session raised the number of paid Commissioners in Lunacy to 8 from 6 which had been the authorised number since 1845. Between that year and 1912 the number of mental cases under their care has risen from 25,000 to 120,000. The principle, by the way, of giving these posts to women has been approved, though, it is stated, the Lord Chancellor appointed no women on this occasion owing to the temporary character of the Bill.

### DR. BOND'S RECORD.

The one medical superintendent who has been added to the Lunacy Commissioners has a record of mental hospital work which it is interesting to recall. Dr. Bond graduated M.D. at Edinburgh University in 1895, and D.Sc. in 1898. His former appointments include that of medical superintendent of the Ewell Epileptic Colony, and senior assistant medical officer at Bexley Mental Hospital. He has written much on mental disease, on which he has been lecturer at Middlesex Hospital.

### THE KENSINGTON PROTEST MEETING.

The medical men of the Kensington and Chelsea divisions of the British Medical Association met at Hammersmith on Tuesday under the presidency of Dr. Fred J. Smith, and the upshot was the almost unanimous appointment of a Reform Committee, with the object of securing an amending Act in which the "Six Point Programme" of the Association shall be safeguarded in any scheme of National Insurance. Dr. Smith sharply criticised the policy of the Council of the Association, and said that they must use every legitimate endeavour to get rid of those members of the Council who approved of the Act. After Mr. Percy C. Raiment had outlined a scheme for the Reform Committee, Mr. Milton Townsend proposed and Mr. William S. Lee seconded a resolution approving of its formation. Interruptions of various kinds then began, Dr. Crawford Thomson and Mr. D. Aloysius O'Sullivan being followed by Dr. Burnhill, who declared that their object was not to weaken by disunion, but merely to stiffen the backs of those in power. Dr. Beckett-Overy disagreed with the prevailing censure of the Council, but was considerably interrupted in his speech, and the resolution was then put and carried almost unanimously. Every member of the Reform Committee pledges himself to decline medical service under the present Act unless it is amended on the "Six Point" basis.

### THE FLORENCE NIGHTINGALE STATUE.

All who have followed the history of the Florence Nightingale Memorial Fund, from its threatened failure to its present flourishing condition, which succeeded The Hospital's emphatic statement of the case, and which followed our appeal for further subscriptions, will be glad to learn that the Office of Works has sanctioned Waterloo Place as the site for her statue. According to a recent proposal the Florence Nightingale statue will be enhanced by the removal from the quadrangle of the War Office of the statue of Sidney Herbert, who was Secretary of State for War during the Crimean campaign, and who, indeed, was responsible for sending Miss Nightingale to the front. There is no doubt that the placing of these two statues in relation to each other will awake many reflections in any passer-by who has the smallest inkling of the condition of our sick soldiers before Florence Nightingale took up their cause.

### QUEEN ALEXANDRA SURPRISES A HOSPITAL.

The West Norfolk and Lynn Hospital on Thursday last week received a surprise visit from Queen Alexandra, who with Queen Amelia arrived unannounced at 3.30. Her Majesty, who knows this hospital perhaps better than any other, having visited it frequently of late years, had no difficulty in finding the door of the matron's room. Miss Swain, the matron, thereupon conducted their Majesties through the wards, many of the patients in the children's and men's and women's wards receiving boxes of chocolates from the visitors. As a matter of fact almost to a day a year ago the Queen, accompanied by the Prince of Wales, surprised the hospital in the absence of the matron, when the deputy-matron, Miss Austin, had the honour of receiving her Majesty.

### STAFF CHANGES AT BART.'S.

The resignation is announced of Dr. Norman Moore, senior physician to St. Bartholomew's Hospital, after long years of distinguished service to that institution in many capacities. Dr. Moore served the unusually protracted term of nineteen years as assistant physician, reaching the full physicianship in 1902. He was also for seventeen years Warden of the College, and for sixteen years lecturer on pathology in the medical school, before promotion to lecturer on the Principles and Practice of Medicine. He also served both hospital and school in several capacities in the seventies, before his election to the visiting staff. Now that Dr. Moore is leaving Bart.'s, it may be hoped he will have more leisure for those scholarly essays on the history of medicine and similar subjects which have spread his reputation far beyond the limits of his medical *alma mater*. The vacancy thus created will doubtless be keenly contested; for there has been a stagnation of promotion on the medical side lately at St. Bartholomew's, and several of the young physicians are thirsting for a chance to show their capabilities. The late medical registrar held that post for, we believe, eight or nine years; and it is no secret that his chances of promotion are thought to be greater than those of any rival candidate. There is also a vacancy at St. Bartholomew's for a surgical registrar, for which competition is also very keen. In any event, these vacancies mean promotion all down the line; and St. Bartholomew's is fortunate just now in having an abundance, almost indeed a plethora, of highly eligible men waiting for promotion.

### INFIRMARY PATIENTS SLEEPING ON THE FLOOR.

The medical officer to the Ipswich Workhouse Infirmary, Mr. J. R. Staddon, has just called attention to the lamentable condition of overcrowding which he says prevails in that institution. So full have the wards been that, according to his own account, not only have extra beds been placed in the large wards but "for the last month or two, patients have been sleeping on the floor." "If an epidemic of sickness broke out," Mr. Staddon continued, "we would be in a dilemma." The nature of this dilemma is not very pleasant to think about, but the interesting thing is why the workhouse committee has not been asked to consider this unfortunate state of things before. To let it go on, apparently unconsidered, "for a month or two" will seem to many to be courting disaster and to reflect upon the management.

### DR. SOPHIA JEX-BLAKE AND HER WORK.

On Sunday last the leader of the pioneer movement in the seventies for the medical education of women, Dr. Sophia Jex-Blake, died at her Sussex home at the age of seventy-one. She began life as a mathematical tutor at Queen's College, London. A visit to America attracted her attention to the efforts made by the late Dr. Elizabeth Blackwell for the medical education of women, that lady, herself qualified, having been appointed in 1865 lecturer on hygiene at the New York Infirmary and College for Women. Miss Jex-Blake thereupon decided to adopt medicine as her profession and studied under Dr. Lucy Sewall, of Boston. On returning to England in 1868 Miss Jex-Blake found that, though Miss Elizabeth Garrett had got her name placed on the British Medical Register in 1865, no British school would admit women to its classes. In 1869 she turned to Edinburgh, and when that University refused to make an exception in favour of one lady, she produced so promptly a body of applicants that separate classes for women medical students no longer could be refused. Their success in the class lists excited opposition, and an application to study in the wards of the Edinburgh Royal Infirmary produced much opposition on the part of the students. The Senate, therefore, was induced to reject a petition signed by 9,000 women to complete their studies, and in 1872 Lord Gifford allowed the action to be brought by the ladies against the Senate, which decision, however, was rejected on appeal. Miss Jex-Blake therefore gave up Edinburgh as hopeless and in 1874 transferred her attack to London, where, as the result of her and others' efforts the London School of Medicine for Women was connected with the Royal Free Hospital in 1877. There medical education and clinical experience was won at last, and in 1876 Mr. Russell-Gurney's Bill enabling British examining bodies to include women among their candidates was passed. In 1878 Dr. Jex-Blake, as she had become then, started practice in Edinburgh and opened a dispensary for women and children, which became a cottage hospital in time. Her final triumph was gained with the opening in 1886 of the Edinburgh School of Medicine for Women, of which Dr. Jex-Blake was herself the founder and first dean. Her book on "Medical Women" was first published in 1872.

### LADY ALMONER'S APPOINTMENT.

The proved necessity of appointing almoners who have had a specific training for their work has been so well recognised that it is interesting to note the appointment of Miss Grahame as almoner to the Royal Waterloo Hospital for Children and Women. Miss Grahame, who has just taken up her duties, has been trained by the Hospital Almoners' Council, the inaugural meeting of which was noted in The Hospital of December 2, 1911 (page 220).

### OVERCROWDING A COUNTY MENTAL HOSPITAL.

An inspector of the Local Government Board has just visited Notts County Mental Hospital at Radcliffe-on-Trent, in view of the application of its authorities for leave to borrow money for the large extension which they desire owing to the present overcrowding of the institution. In answer to a question, the Medical Superintendent, Dr. S. Lloyd Jones, said that the number of mental cases had increased. Last year apparently the number of admissions was exceptional. At present 485 patients are on the register, while, according to Dr. Jones' reported statement, the hospital accommodation is only for 452. Besides this, 64 patients are boarded out. The proposed extension, for which £29,300 is asked for, would increase the accommodation to 600. Mr. L. Maggs, the county architect, proposes, it is understood, to add two wings to the institution. It would be interesting to learn to what extent the pressure on the hospital's accommodation has been due to recurrent insanity.

### COUNTRY CLERGY AND SECRET REMEDIES.

Our recent argument for a Royal Commission on Quackery has brought us the interesting letter which we publish elsewhere under the above title. Anything which tends to break down the newspaper boycott to which that admirable work, "Secret Remedies," was subjected is worth reading, whether it comes from a London weekly or whether, as in this case, it is found in a small country parish magazine. The Vicar, Mr. C. L. Marson, as the writer of the letter points out, is not adopting the "usual" course in explaining to his parishioners the worthlessness of many patent medicines, but can it be true, as our correspondent alleges, that the clergy are importuned by "pill proprietors" to "push" the sale of their wares? The point is well worth raising, and all who remember how Jude Fawley was induced to advertise Physician Vibert's nostrums in Mr. Thomas Hardy's last, and in certain senses greatest, novel will realise how much an unscrupulous clergyman, who shared the general poverty of his profession, could do to increase the sale of patent medicines in his parish.

### MENTAL PATIENTS IN A GENERAL HOSPITAL.

The past year at the Edinburgh Royal Infirmary has been important as seeing the highest ordinary income ever recorded in the history of the hospital. Another interesting fact is the agreement made at the end of 1909, between the hospital's managers and the Edinburgh Parish Council, whereby a limited number of incipient mental cases were received from the Council in a ward which the hospital agreed to set apart for this purpose. The experiment has been continued, on the advice of the physician in charge of the ward, who, while admitting that the conditions are imperfect, has advised that the arrangement should be continued until the Parish Council has secured the special institution which, it is understood, has been the aim, or hope, of its medical officer. The Edinburgh Royal Infirmary has had to refuse the Council's request for extended, and still further, accommodation.

### PARTY POLITICS AND THE INSURANCE ACT.

If there is one fact which has become clearly apparent during the past week or two, it is that the Queen's Hall meeting and its after-results have given the Government a bad fright. Last Sunday, for instance, a threat was issued through the *Observer* that, should the opposition of the doctors continue, the contributory portion of the Insurance Act would be dropped and the deficiency made good out of taxation. This ingenious piece of bluff may be good party tactics, but it need scarcely be said that it will not affect the attitude of the profession. The amounts allocated under the present Act are insufficient to pay the doctor a " living wage." If those amounts are to be supplemented so that remuneration becomes reasonable, well and good; but it is no concern of the doctors how the money is obtained. It is worth while to note in passing that the same *ballon d'essai* in the *Observer* foreshadows as part of the amended scheme State subsidies in aid of hospitals and State control as a corollary. That in itself is a big proposition, and one the announcement of which in this casual way scarcely does justice to the magnitude of the issues involved. The production of the State-control bogey at least shows that the discontent among hospital managers with the provisions of the Act is beginning to make an impression.

### ATTITUDE OF THE PARTY PRESS.

If any further evidence is needed to show how much the minds of the authorities are exercised by the revolt of the hospitals and of the doctors, it is supplied by a study of the Ministerialist Press. These party organs are especially distinguished by the assiduity with which they foment any and every kind of strike in the industrial world; and it might be supposed that their sympathy would also be enlisted on behalf of the doctors. Not a bit of it; no words are bad enough to describe the attempts of the profession to avoid exploitation and sweated service. The *Daily News*, for example, waxes sarcastic and indignant against the medical profession because two children died near Christmas without medical attendance. The parents were too poor to pay medical fees, and the *Daily News* calls this the breakdown of the present system. Has the editor never heard of voluntary hospitals or of the Poor Law infirmaries? He speaks of neglect, as if the case showed neglect on the part of the doctor who was called in after death took place: surely the neglect was on the part of the parents in omitting to take their child to the nearest hospital for free treatment! After a cheap sneer at Sir James Barr, he has to admit that the Insurance Act would not have provided these children with medical attendance; though he does not add, as he might do, that it will also undermine the voluntary hospital system, whereby they could have been succoured. The editor of the *Daily News* recommends Sir J. Barr to study the reports of these inquests. Before he again attempts to stir up prejudice and passion, in order to beg the question whether or not the doctors have a right to bargain with the State for a decent wage, he might well himself devote a little time to the other two inquests at Hackney on which we commented last week (The Hospital, January 6, 1912, p. 351). He will then be in a better position to judge the value of medical attendance of the kind which the Insurance Act is likely to provide.

### SPECIAL DEPARTMENTS AT GUY'S.

The authorities at Guy's Hospital are moving with the times in taking steps to secure that the recently established special departments at that hospital shall be in charge of specialists who have been specially trained in their work. The genito-urinary department, as we stated some time ago, has been placed in charge of Mr. Thompson, who has studied this specialty at Paris. Now the Governors invite applications for the post of surgeon in charge of the orthopædic department. The successful applicant, it is particularly stated, must go abroad for a definite period to familiarise himself thoroughly with Continental methods. Afterwards the department is to be placed wholly under his control. At present the orthopædic department at Guy's exists in embryo only. There are no exercise rooms, there is no trained instructor in physical drill, and there is not even, so far as we are aware, a single piece of apparatus suitable for the complicated muscle movements now demanded in the after-treatment of orthopædic cases. The department has hitherto been in charge of one of the junior assistant surgeons, who has not had the benefit of Continental or American teaching in orthopædics. In the circumstances the decision of the Governors to bring this department in line with the genito-urinary and aural departments—which at Guy's may claim to be properly-equipped and well-managed special departments—will be appreciated by everyone who is interested in the development of the South London Institution.

### A HOSPITAL'S WORK IN YORK.

The Working Men's Fund, which has done so much for the York County Hospital, has recently lost its founder Mr. G. Briggs, J.P., who has left the city. All people who understand the importance of these funds will appreciate the House Committee's regret at his departure. Mr. Briggs was the fund's first president, and for eight years has been a member of the House Committee of the York County Hospital. An unusual experience attended the recent quarterly meeting; for the second quarter in succession no donation of over £20 has been received, and there have been no contributions at all save those from the workpeople's collections. This fact has peculiar significance when regarded in the light of the departure of the founder of these collections. Nothing could show better how prescient were those who urged and worked for the inception of these funds.

### A YEAR'S WORK IN NEW BUILDINGS AT MANCHESTER.

The new premises occupied by the Manchester Ear Hospital have lent particular interest to its latest report, which shows an increase in the work done. There is an eloquent proof of the pressing need for further accommodation. The new wards and the new theatre have been well filled—so well filled indeed that more than double the number of operations have been performed than was possible in the old quarters. The House Committee, enforced by those of its members who are also lady visitors, has secured, says the report, more economical management. The hospital, apparently, has been worked to its fullest capacity, and the consensus is that its expansion has produced satisfactory results all round.

### NEW APPOINTMENT TO THE SATURDAY FUND.

The result of the ballot for a new assistant organising secretary to the Hospital Saturday Fund has resulted in the election of Mr. Edwin P. H. Penn. There were no fewer than thirty-eight applicants for the post. Mr. A. W. Davis, the secretary of the Fund, is of opinion that the total for the past twelve months will show an increase of £2,500 over previous years. At the time of going to press this has not yet been confirmed. Two contributing firms apparently have decided to discontinue their subscriptions on account of the passing of the Insurance Act.

### EXTENSION SCHEMES AND THE INSURANCE ACT.

The Cumberland Infirmary is having to face the problem of increased cost of maintenance, consequent upon the extension scheme that was completed last year. It will be remembered that the new Nurses' Home, or the Barnes wing, as it has been called gracefully after Dr. Barnes, was the first part to be finished, but with this and other improvements behind it the new year is raising the question of how far it is possible to increase the number of subscribers. We draw attention to this as showing that it is now or never for the hospitals to unite and to follow the leadership of the British Hospitals Association in taking a common action in regard to the Insurance Act.

### NEW PRESIDENT OF THE SEAMEN'S HOSPITAL.

Prince Louis of Battenberg, Admiral of the Fleet, is the new President of the Seamen's Hospital at Greenwich. The fact has a special interest, as, since the death of the Duke of Saxe-Coburg and Gotha (the Duke of Edinburgh), no sailor, oddly enough, has held this post, the chief office of the oldest marine charity. The appropriateness of Prince Louis' appointment is therefore strongly felt, and by the way, one of his early duties will be to preside at the dinner of the London School of Clinical Medicine, which will take place on Friday, March 1.

### THE KING EDWARD MEMORIAL AT WORTHING.

The new out-patient buildings that have been erected during the autumn at the south-west corner of Worthing Hospital are practically finished, and the public will shortly, in the new year, see them opened. Designed by Mr. A. Morris Butler, and erected by Mr. George Baker, junior, the total cost of the work is expected to be, including the new gates, £3,000. Thanks to Alderman E. E. C. Patching, J.P., between one and two thousand have been raised. No adequate description is possible without the plans, but the inevitable central-hall plan has of course been adopted. In February it is hoped the Duke of Norfolk will find time to attend the opening.

### THE SAXON SNELL PRIZE.

The Council of the Royal Sanitary Institute is offering a prize of fifty guineas and the silver medal of the Institute for an essay on "Suggestions for Improvements in the Ventilating, Lighting, Heating, and Water-supply Appliances and Fittings for an Operating Room and its accessory rooms for a General Hospital of 400 Beds (no Students)." The funds enabling them to do so were left by the late Henry Saxon Snell, who founded the prize, which is to be awarded every three years "to encourage improvements in the construction or adaptation of sanitary appliances." The general conditions state the essay must not contain more than 5,000 words and must be delivered on or before August 30, 1912, to the secretary of the Institute at 90 Buckingham Palace Road, S.W. Another condition states that two competitors of different professions or crafts may join in sending an essay and plans. It is interesting to note that so purely an institutional subject as improvements in an operating theatre has been chosen, and the younger generation of hospital architects may find food for thought, and the hope of reward, in attempting to answer the questions it raises. Perhaps some will think that hospital theatres have received almost more than their due share of attention, and that its selection by the Institute is possibly explicable by the immensely wide range of sanitary subjects that is suggested by a surgical theatre of the latest type. Only the other day we were commenting on one of the latest theatre-units that have been built, that namely at the North Riding Infirmary.

### WELL-KNOWN BIRMINGHAM PHYSICIAN RETIRES.

Dr. Robert Saundby, F.R.C.P., senior honorary physician to the Birmingham General Hospital, one of the ablest physicians of the day, has just resigned his post after twenty-seven years. Dr. Saundby is an M.D. of Edinburgh and an LL.D. of St. Andrews, and President of the British Medical Association 1911-12. He came to Birmingham in 1876, when he took up the post of pathologist at the General Hospital. In 1885 he became honorary physician. Among his numerous publications his "Medical Ethics," which appeared in 1907, has gone through two editions. The recent meeting of the Board of Management, over which Mr. J. C. Vaudray presided, accepted Dr. Saundby's resignation with many expressions of regret.

### THE NEW DISPENSARIES IN ROME.

A correspondent sends us the following concerning the Hygienic Exhibition at Rome, which was opened on December 30 at the Castel S. Angelo: "Special prominence is given to anti-tuberculosis prophylactics; and, in particular, the appositely-constructed dispensary, which will remain open definitely after the closing of the exhibition. It is intended not only for the cure and registration of cases of tuberculosis, but also for the recovery of the sick in the neighbourhood of Castel S. Angelo. There is a small sanatorium annexed to the dispensary, which is provided with a refectory, a bath saloon, a spacious verandah, and a small garden which the poor patients can cultivate in their hours of leisure. Hygienic education will be provided for the convalescents both in the form of conferences and pamphlets. This will be the first of a series of dispensaries which will be established in various quarters of Rome."

### "TREE DAY" AT THE MANCHESTER CHILDREN'S HOSPITAL.

Wednesday last week was kept as "Tree Day" at the Manchester Children's Hospital at Pendlebury, and six huge trees, lighted by 216 coloured electric lamps, were placed in the centre of each of the six wards. The Borchardt Ward Christmas tree, portraying "Fairyland," reflected great credit upon Sister Storey and her nurses, whilst the Christmas tree representing the four seasons drew many encomiums for Sister Foulkes and her staff in Wrigley Ward. In the Heywood Ward Sister Stower had provided a Japanese tree, which formed a dressed representation of Japan. Packets and toys lying underneath the trees were distributed in Heywood Ward by Mr. J. Howson Ray, F.R.C.S.; in the Holden Ward by Mr. Wm. Buckley, M.P., Chairman of the House Committee; in the Borchardt Ward by Mr. John Dendy, Hon. Secretary; in the Victoria Ward by Mr. F. Simpson; in the Wrigley Ward by Mr. A. K. Armitage, J.P.; in the Liebert Ward by Mr. Rayner, F.R.C.S. The following Patrons, Governors, and members of the staff took part in the proceedings in the wards: The Mayor and Mayoress of Salford, the Chairman of the Board Mr. H. A. Heywood, Dr. H. R. Hutton, Dr. C. Christopher Heywood, Chairman of the Medical Committee, Mr. Charles Roberts, F.R.C.S., Mr. Wilson H. Hey, F.R.C.S., Dr. C. P. Lapage, the resident Medical Officers, and the Matron, Miss Renant. It is interesting to note that at this hospital Christmas Day itself pales before "Tree Day," which is quite an institution in itself.

### AN OBJECT-LESSON FROM PERU.

Mr. Percy Martin's "Peru of the Twentieth Century," recently published by G. E. Arnold, is, among many other things, something of an object-lesson to those who are remiss in their support of medical charitable institutions in the United Kingdom. The generosity of the Spanish-American towards his poorer brother is an example to be imitated. Mr. Martin says: "In several of the cities and towns there are found institutions which are conducted and entirely supported by private charity. The Lima Benevolent Society is an institution which has effected a great amount of good. Its revenues exceed 1,000,000 dollars annually, and out of these the Society entirely supports the Second of May Hospital, a modern institution which accommodates a thousand patients of both sexes. It also partially maintains the Santa Ana Hospital for Women, which was founded in 1549 by the first Bishop of Lima, Monsignor J. Geronimo de Loaiza, while it assists with finances and controls the management of the Maternity Hospital, the School of Midwifery, and the Military Hospital of San Bartolom ." The fact that the cost of living in Peru is decidedly expensive does not prevent many charitable medical institutions from being vigorously supported.

### SPECIAL GIFT TO THE TOTTENHAM HOSPITAL.

Sir Ernest Cassel, whose gift of £50,000 to the hospitals has been recorded already in these columns, has, it is announced, further allocated the special sum of £700 to the Prince of Wales General Hospital, Tottenham. It should be remembered that this hospital's share in Sir Ernest Cassel's generous gift to King Edward's Hospital Fund was £800. All who know the work and claims of the Prince of Wales General Hospital will join in congratulating its director, Mr. F. W. Drewett, on this important accession to its funds.

### OLDEST HOSPITAL IN PARIS REBUILT.

The Hôtel-Dieu, which is not only the most ancient hospital in Paris but perhaps in Europe, is about to be reconstructed. It was founded by St. Landry about the years 651-656. It has undergone considerable change for the better since the Revolution, but when the cholera desolated Paris in 1832 we believe we are right in recalling the fact that only one patient survived out of the first 600 received into this hospital and only five out of the first 1,000. The scheme of reconstruction, which is very extensive, is expected to cost about £60,000, and while modernising the interior it is understood that care will be taken not to interfere with the architectural style of the building.

### WARDMAIDS' PROPER BEDTIME.

In most houses and institutions the maids have to begin work at 6.30 in order to do all that is necessary before an eight o'clock breakfast. We see, therefore, that they should be in their bedroom by 9.30 in the evening, yet we very much doubt whether they get there before 10 P.M. in most houses, and frequently it is nearer 10.30. In every household in which the domestic servant is expected to be down by 6.30 A.M. and in every institution where wardmaids are required to do similar work, the work should be so arranged that she goes to her room at 9.30 P.M. If these full hours be not given the girl will probably always be tired in the morning, or the extent of her ablutions and personal care must suffer, an undesirable thing in one who is continually doing dirty work.

### SPECIAL FURNITURE FOR HOSPITAL PATIENTS.

In a private house the patient can readily be kept in the upright position by putting a pillow under his knees and then tying the corners by bandage or tape leaders to the head of the bed above the patient's shoulders. In an institution where such cases are likely to occur frequently, special furniture may be considered necessary. Three things must be considered: the stretcher, the operating-table, and the bed. All of these must be made so that the patient can recline with his body inclined at an angle of 60 degrees with the horizontal. In the first the stretcher may be replaced by a carrying-chair inclined backwards at the necessary angle. The operating-table should be made so that the part that is raised includes the area on which the back lies, as well as the part under the shoulders and head; the operation can then be performed in this position, with the lower limbs horizontal, and the patient lifted back into the inclined chair. Besides these, some appliance is needed for the bed. This takes the form of a rest triangular in section, which goes beneath the mattress under the knees of the patient. By means of these three appliances the patient can be kept upright from the time when the diagnosis is made until he is out of danger, and by their constant use the results of acute appendicitis cases would probably be considerably improved. Lately several institutions have adopted special appliances, and we shall be glad to receive reports, together with illustrations, regarding the apparatus found most effective and convenient in hospital wards.

### DANGER FROM THE DUSTBIN.

The care of the dustbin is the goal to be aimed at in controlling fly pests in our large cities. No doubt of late years much good has been done in improving dustbins, but it is necessary still to keep a vigilant eye open. Small dustbins that can be lifted on the shoulder and carried straight to the cart should be used. A large bin means that the dustman must shovel out the refuse into his basket, and in so doing he soils the ground and favours the breeding of flies. There is a great temptation to buy a large, strong bin; there is no danger of it being overfilled, supposing the amount of refuse should suddenly increase for some reason; also it is made of stronger material and is not so quickly worn out by the rough usage of being emptied. If, however, this results in the bin becoming so heavy that the man cannot lift it, the economy is a false one in the end. The other important point is to urge on local authorities the need of a daily removal of the refuse.

### THE INCREASE OF FLAT LIFE.

While these things can be well looked after in a private house, where the owner is fully alive to their importance, or in an institution where there is someone responsible for such things, there are possibilities of marked shortcomings in the case of blocks of flats, which are becoming increasingly common nowadays. In these the refuse disposal is entirely in the hands of the porter or caretaker. If he is an efficient man no doubt it is well done, but if he is ignorant or careless great infection of soil near the ground-floor flats may occur. There is a natural tendency to collect the refuse of the whole block at one corner, so that the occupants by one flight of steps get the full benefit of the bins for the whole number. Again, what is everyone's business is no man's business, and even if one tenant complains, his complaint must go to the agent or landlord, as he has no direct authority in the case. It is, therefore, imperative that everyone living in a flat should walk round at the back occasionally, and see that the refuse disposal is well carried out. In this case the example of institutions is one that ought to be followed far more carefully in private life.

### THIS WEEK'S DRUG MARKET.

Few changes in the prices of drugs have occurred during the week, and business on the whole has been quiet, although there has been a fairly good inquiry, which may lead to business. Prospective buyers of cod-liver oil are awaiting reports from Norway as to the early results of the fishing, which begins about this time; given favourable weather, the fishing promises well. Last season's oil is again quoted lower, but practically no business is being done. Quicksilver has been slightly reduced in price since the beginning of the year, but the reduction is not sufficient to justify a fall in the price of the salts of mercury. Carbolic acid still tends towards higher prices. Oil of cloves is again slightly cheaper. Apiol is dearer. Buchu leaves are likely to be higher in price, as the tax on the collection of the leaves on Crown lands in South Africa has been advanced by 1s. per lb.

